# Gluten-Free Bread with Cricket Powder—Mechanical Properties and Molecular Water Dynamics in Dough and Ready Product

**DOI:** 10.3390/foods8070240

**Published:** 2019-07-03

**Authors:** Przemysław Łukasz Kowalczewski, Katarzyna Walkowiak, Łukasz Masewicz, Olga Bartczak, Jacek Lewandowicz, Piotr Kubiak, Hanna Maria Baranowska

**Affiliations:** 1 Institute of Food Technology of Plant Origin, Poznań University of Life Sciences, 60-624 Poznań, Poland; 2 Department of Physics and Biophysics, Poznań University of Life Sciences, 60-637 Poznań, Poland; 3 Students’ Scientific Club of Food Technologists, Poznań University of Life Sciences, 60-624 Poznań, Poland; 4 Chair of Production Engineering and Logistics, Poznan University of Technology, 60-965 Poznań, Poland; 5 Department of Biotechnology and Food Microbiology, Poznań University of Life Sciences, 60-627 Poznań, Poland

**Keywords:** gluten-free bread, edible insects, protein enrichment, rheology, texture, ^1^H NMR, water behavior, water activity

## Abstract

Published data indicate that cricket powder (CP) is a good source of not only protein, fat and fiber, but also minerals. Due to the fact that this product naturally does not contain gluten, it is an interesting addition to the enrichment of gluten-free foods. This paper is a report on the results of starch substitution with CP (at 2%, 6% and 10%) on the properties of dough and bread. The rheology of dough and the texture of the final product were studied. While the changes caused in the dough by the introduction of CP were not pronounced, the bread obtained from it was characterized by significantly increased hardness and improved consistency. Analyses of water behavior at the molecular level with the use of ^1^H Nuclear Magnetic Resonance (NMR) indicated that CP altered both the bound and bulk water fractions. Moreover, examination of water activity revealed a decreased rate of water transport in samples of bread that contained CP. These results indicate improved availability of water to the biopolymers of bread, which likely plays a role in shaping the textural properties of the product.

## 1. Introduction

An increasing number of patients with celiac disease has led to increased interest in gluten-free (GF) products. Celiac disease is characterized by permanent gluten intolerance which, in turn, results in histopathological changes within the mucosa of the small intestine [1]. The only effective way to combat it is strict adherence to the GF diet [2,3]. It is estimated that about 1% of the population suffers from this disease [4,5,6,7]. Although the effectiveness of a gluten-free diet has not yet been proven for other diseases except celiac disease, it is also often recommended by doctors in other disease entities, such as non-celiac gluten sensitivity, Hashimoto’s disease and irritable bowel syndrome [8]. Thus, the GF product market continues to grow.

Gluten is responsible for the retention of gases in dough, as well as for giving dough the right consistency. GF bread is characterized by structure and texture that is generally perceived as unattractive. In order to improve the properties of bread, including its aroma, additives, for example hydrocolloids, are used [9,10,11,12]. Moreover, GF bakery products are characterized by improper nutrient composition that results from the substitution of gluten containing flour with alternative starchy raw materials. Compared to traditional cereal products, GF breads have significantly lower nutritional value, especially in terms of decreased content of fiber, minerals and protein [9,13,14]. The additives used for the production of GF bread can supply the missing nutrients. Among such additives, edible insects can be distinguished.

In Africa, Latin America and Asia, edible insects have been known as a foodstuff for years [15,16]. As reported by the United Nations Food and Agriculture Organization, more than 1900 species of insects are eaten worldwide, including crickets, meal larvae, ants, grasshoppers and flies [17]. Research results published so far indicate that crickets, as well as cricket powder obtained from them, are a valuable source of protein, fat and minerals [18,19,20]. They also contain bioactive compounds [21,22]. Efforts have thus been undertaken to introduce them to the production of many food products [23,24,25]. To date, however, the impact of cricket powder (CP) on the characteristics of GF dough and the texture of GF bread has not been described. Therefore, the aim of the work was to assess the influence of cricket powder on the rheological properties of dough and the resulting texture of GF bread. Furthermore, water behavior in the tested bread samples was investigated with the use of low-field Nuclear Magnetic Resonance (NMR).

## 2. Materials and Methods 

### 2.1. Materials 

Corn starch was purchased from Glutenex (Sady, Poland), potato starch from PPZ Trzemeszno sp. z o.o. (Trzemeszno, Poland), guar gum from Limpio Chem LLP (Gujarat, India), pectin from Silvateam S.p.a. (via Torre, Italy), yeast from Lesaffre Polska (Wolczyn, Poland), sugar from Pfeifer & Langen Polska S.A. (Środa Wielkopolska, Poland), salt from Ciech Soda Polska S.A. (Janikowo, Poland) and rapeseed oil from ZT ‘Kruszwica’ S.A. (Kruszwica, Poland). The edible cricket powder was obtained from Crunchy Critters (Derby, United Kingdom). All chemicals and reagents used were of analytical grade.

### 2.2. Production of Bread

The recipe for reference gluten-free bread was as follows: 200 g corn starch, 50 g potato starch, 4.25 g guar gum, 4.25 g pectin, 15 g yeast, 5 g sugar, 4.25 g salt, 7.5 g rapeseed oil and 275 g distillated water [26]. Dough was prepared using the straight dough method. All the compounds, except oil, were mixed together with the use of KitchenAid mixer (model 5KPM5EWH, KitchenAid, Benton Harbor, MI, USA) for 2 min at a speed of 70 rpm, then oil was added, and mixing was continued for 6 min. Next, the dough was fermented in a fermentation chamber for 20 min (temperature 35 °C, relative humidity 85%) and punched. Each sample of dough was divided into two parts (280 g each) and placed in baking forms. The final fermentation was carried out for 15 min at 35 °C. Prepared dough was baked at 230 °C for 30 min (MIWE Michael Wenz GmbH, Amstein, Germany). Afterwards, the obtained breads were left at room temperature for 2 h to cool down, weighed and packed in polypropylene pouches. In the test samples, total starch was replaced with CP in three different quantities of 2%, 6% and 10%; the amounts of other components were unchanged. Reference dough and bread were denoted in the text as DB and RB, respectively. The dough samples containing cricket powder were named DCP2, DCP6, and DCP10 and the bread samples obtained from them were named BCP2, BCP6, and BCP10, respectively.

### 2.3. Rheological Properties of Dough

Viscoelastic properties were determined with the RheoStress1 rheometer (Haake Technik GmbH, Vreden, Germany) in controlled deformation mode (CD) with deformation set to 0.5%. Mechanical spectra were obtained within an angular velocity range of 0.1–100 rad∙s^−1^. The diameter parallel plate measurement geometrics (PP35 Ti) were 35 mm with a 1.0 mm gap. Complex viscosity (η*), storage modulus (G’), and loss modulus (G’’) were determined. The Ostwald de Waele equation (η*) and the power law equations (G’ and G’’) were used to model the obtained spectra.

(1) $$\eta^{*}=\;K^{*}\times \omega^{n^{*}-\;1},$$

where η* is complex viscosity (Pa∙s), K* is consistency index (Pa∙s^n^), ω is angular velocity (rad∙s^−1^) and n* is flow behavior index (-).

(2) $$G\prime =\;K\prime \times \omega^{n^{\prime }},$$

where G′ is storage modulus (Pa), K′ is the equation constant (Pa∙s^n^), ω is angular velocity (rad∙s^−1^), *n*′ is the equation constant (-).

(3) $$G\prime \prime =\;K\prime \prime \times \omega^{n^{\prime \prime }},$$

where G″ is loss modulus (Pa), K″ is equation constant (Pa∙s^n^), ω is angular velocity (rad∙s^−1^), *n*″ is equation constant (-).

### 2.4. Texture Analysis

Texture profile analysis of bread was performed with a TA.XTplus texture analyzer (Stable Micro System Co. Ltd., Surrey, England) equipped with a 5 kg load cell [27]. Each sample was compressed twice with a cylindrical plunger probe with a 35 mm diameter. The test parameters were as follows: 10.0 mm s^−1^ pre-test speed, 5.0 mm s^−1^ test speed, 5.0 mm s^−1^ post-test speed, and 40% strain. Bread loaves were cut into slices (25 mm thick each and ends were discarded) and used to evaluate hardness, springiness, cohesiveness, chewiness and resilience. Texture analysis was repeated 15 times for each sample.

### 2.5. NMR Relaxometry

NMR measurements were performed according to Baranowska et al. [28]. Crumb or dough samples of 1.5 cm^3^ were placed in measuring test tubes and sealed using Parafilm^®^ (Bemis Company, Inc., Joplin, MO, USA). Measurements of the spin–lattice (T_1_) and spin–spin (T_2_) relaxation times were performed using a pulse NMR spectrometer MSL30 operating at 30 MHz (WL Electronics, Poznań, Poland). The samples were measured at 21.0 ± 0.5 °C. The inversion-recovery (180−t−90) [29] pulse sequence was used for measurements of the T_1_ relaxation times. Distances between RF pulses (*t*) were changed within the range from 80 to 130 ms and the repetition time was 10 s. Each time, 32 free induction decay (FID) signals and 119 points for each FID signal were collected. Calculations of the spin–lattice relaxation time values were performed in CracSpin program using the ‘spin grouping’ approach. Marquardt’s method of minimization was used for fitting multiexponential decays. Standard deviation was used to determine the accuracy of the analysis of relaxation parameters. Time changes of the current value of the FID signal amplitude in the employed frequency of impulses were described by the following formula:

(4) $$M_{z}\left(t\right)=M_{0}\left\{1-2\exp\left(\frac{-t}{T_{1}}\right)\right\},$$

where M_z_(*t*) is the actual magnetization value and M_0_ is the equilibrium magnetization value.

Magnetization recovery was determined monoexponentially, which means that the system relaxes with one T_1_ spin–lattice relaxation time. Measurements of the spin–spin (T_2_) relaxation times were taken using the pulse train of the Carr–Purcell–Meiboom–Gill spin echoes (π/2–TE/2–(π)*_n_*) [29]. The distance (τ) between 180 RF pulses amounted to 1 ms. The repetition time was 10 s. The number of spin echoes (*n*) amounted to 50. Eight accumulation signals were employed. To calculate the spin–spin relaxation time values, the authors applied adjustment of values of the echo amplitudes to the formula [30]:

(5) $$M_{x,y}\left(\tau \right)=M_{0}\sum_{i=1}^{n}\;p_{i}\exp\left[\frac{-\tau }{T_{2i}}\right],$$

where M_x,y_(τ) is the echo amplitude, M_0_ is the equilibrium amplitude, and p_i_ is the fraction of protons relaxing with the T_2i_ spin–spin time.

The calculations were performed using the dedicated software by application of the non-linear least-square algorithm. The accuracy of the relaxation parameters was estimated with standard deviation. The presence of two proton fractions was determined for all analyzed systems.

### 2.6. Measurements of Water Activity

Analyses of water activity a_w_ in the bread crumbs were conducted using a water diffusion and activity analyzer, ADA-7 (COBRABID, Poznań, Poland), with automatic recording of water evacuation from individual samples [31]. The thickness of the sample placed in the measurement chamber was 5 mm. Before the analysis, the temperature was stabilized at 21.0 ± 0.1 °C. The sample was then dried to the activity of 0.1000 ± 0.0005. The duration of each measurement was 1200 s. Water activity measurement results were used to describe water transport in breads with the use of the following phenomenological model [32]:

(6) $$a_{w}\left(t\right)=a_{r}+\left(a_{0}-a_{p}\right)e^{-V_{D}t}+\left(a_{p}-a_{r}\right)e^{-V_{p}t},$$

where a_w_(*t*) is the temporary water activity value, a_0_ is the initial water activity, a_p_ is the limit water activity (intermediate), a_r_ is the water activity at equilibrium condition (final), V_D_ is the transport rate, and V_p_ is the rate of the surface conduction.

### 2.7. Statistical Analysis

For every test, three independent measurements were taken, unless stated otherwise. One-way analysis of variance was performed independently for each dependent variable. Post-hoc Tukey HSD multiple comparison tests were used to identify statistically homogeneous subsets at α = 0.05. Statistical analysis of the data was performed with Statistica 13 (Dell Software Inc., Round Rock, TX, USA) software.

## 3. Results and Discussion

### 3.1. Dough Rheology

The vast majority of food materials, including dough, exhibit rheological characteristics, which makes it impossible to classify their state as either solid or liquid. Such materials show both elastic and viscous properties [33]. Elastic properties are represented by the storage modulus (G’), which describes the energy temporarily stored in the sample that can be recovered, whereas viscous properties are described by the loss modulus (G’’) that corresponds to the energy used for initiation of the flow that is irrevocably converted into shear heat [34]. Mechanical spectra of gluten-free doughs are presented in Figure S1.

Parameters of power law equations describing the visco-elastic properties of gluten-free dough enriched with cricket powder are presented in Table 1. The fit of the employed models to the experimental data was good, as indicated by the values of coefficient of determination (*R*^2^), which exceeded 0.97. All investigated samples were characterized by the dominance of solid-like behavior indicated by the fact that the values of K’ were greater than K’’. This is typical even for more sol-like materials, for example, starch paste [35]. Replacement of starch by cricket powder in amounts up to 6% resulted only in minor changes in rheological properties of the analyzed dough samples. The only relevant change observed was the decrease in complex viscosity (K*), which was a result of a decrease in both types of mechanical properties (K’ and K’’). Similar values of n*, n’ and n’’, determined for samples RD, DCP2 and DCP6, suggest that a minor decrease in viscosity was observed over a wide range of angular velocity values. This was the only change in the mechanical properties of the dough caused by replacement of starch by cricket powder in those samples. Further increase in the cricket powder to starch ratio in dough resulted in a significant decrease in viscosity along with an increase in all n equation parameters. This involved a stronger decrease in viscosity at higher shear forces compared to other dough samples.

### 3.2. Water Behavior of Dough and Crumb

Low-field NMR is a method used in food analysis since the 1990s. It allow one to measure the spin–lattice T_1_ and spin–spin T_2_ relaxation times, which characterize the molecular dynamics of water in a sample [30,31,36,37]. The parameters of molecular dynamics of water in the dough and crumb of bread were determined on the basis of the ^1^H NMR tests and are presented in Table 2. The presence of two water fractions (bound and bulk) was found, which is a typical result for this type of material [38,39]. With the increase in the amount of starch substituted by CP, a significant decrease in the value of spin–net T_1_ and both components of the spin–spin T_2_ relaxation times was observed. This indicates that CP addition resulted in the decrease in the ratio of bulk-to-bound water fractions. The method of producing CP (roasting and grinding of insects) makes it hydrophobic instead of hydrophilic [40]. The results obtained therefore suggest that the introduction of CP leads to a greater availability of water for the biopolymers in the dough. This has influence on the viscoelastic properties of the dough—a network formed by starch and hydrocolloids (Table 1). The measurements of the relaxation time in the bread crumb show that after thermal processing the amount of bulk water fraction in relation to bound water fraction decreases with increasing amounts of CP additive. In the case of RB and BCP2, the value of the T_1_ parameter was lower by approximately 15% after baking in comparison to RD and DCP2, respectively. The other two breads were characterized by a 20% decrease in the value of this relaxation time. There were no statistically significant changes in the value of the spin–spin relaxation time T_22_ for the crumb samples RB, BCP2 and BCP6 that would result from the presence of CP. At the same time, the comparison of the value of this parameter between dough and the respective crumbs shows a 3-fold decrease for the RB sample and a 2-fold decrease for the BCP10 sample. The fact that the T_22_ time was decreased in all the bread samples compared to the respective dough samples indicates that the baking process resulted in the removal of free water. The water available for biopolymers and hydrocolloids was largely retained in the structure. This can be evidenced by both a relatively small decrease in the T_1_ value for crumbs and dough in individual samples and the absence of statistically significant changes in the value of both components of the spin–spin relaxation time.

There was no effect of the substitution of starch by CP on water activity at equilibrium condition (a_w_) and limit water activity (a_p_) of the crumb (Table 3). The transport rate (V_D_) was lower in samples containing CP than in the reference bread. The transport rate limitation is the result of interactions between water and starch as well as between water and hydrocolloids. This confirms the previous suggestion based on the analysis of relaxation times that CP present in the bread crumb leads to increased availability of water to biopolymers. Also significantly lower was the rate of surface conduction (V_p_) in samples that contained CP. Combined with the data obtained using low-field NMR, this result confirms the previously described changes in the molecular properties of water that are a consequence of the introduction of CP. 

### 3.3. Crumb Texture

As commonly known, water content and activity have effects on the texture of bread. Texture profile analysis was conducted in order to evaluate these changes. The force required to squeeze the food between the teeth is a measure of the hardness, which is responsible for the perception of the freshness of food [41]. As stated in Table 4, the reference bread had the highest hardness and chewiness values. Moreover, the values of these parameters decreased with increasing amount of CP in the formula of the bread. Emulsifiers are used in baking technology to reduce crumb hardness [42]. The softening effect of CP could be connected with the emulsifying properties of cricket proteins. Similar effects on the structure of gluten-free crumbs were previously described by other authors who observed a decrease in crumb hardness after adding natural emulsifiers to dough [43,44,45]. Crumb cohesiveness, a parameter that describes the degree of deformation of the food structure before its breakage, significantly increased with the addition of CP. The increased consistency of the crumb in the case of CP-containing bread samples in comparison to the control sample is undoubtedly a desirable feature. GF breads usually have high susceptibility to fracture or crumbling [46]. Despite the fact that the springiness values did not differ significantly between the tested samples, CP incorporation significantly increased the ability of the crumb to return to its original state after compression, as evidenced by higher resilience values observed in all the enriched bread samples. This could be directly related to the high protein content in CP [18], which significantly affected the formation of the bread texture.

## 4. Conclusions

While substitution of starch with CP may improve the nutritional value of gluten-free bread, it can also cause a number of changes in the properties of both the dough and the final product. Despite the fact that only small changes of macroscopic properties of dough were observed in these rheological analyses, the molecular-level analyses of water contained in the dough revealed that CP increases the availability of water for biopolymers, such as starch or hydrocolloids. This was probably an effect of binding the fat fraction. As a result, significant changes in water dynamics were also observed in the ready bread crumb samples. Moreover, it was shown that the introduction of CP leads to the reduction of hardness of the bread and improves its consistency. While the health-beneficial properties of edible insects are known, more research is needed in order to fully describe the health-promoting properties of bakery products supplemented with cricket powder. 

## Figures and Tables

**Table 1:** The viscoelastic properties of dough.

Sample	K *	n *	*R* ^2^	K’	n’	*R* ^2^	K’’	n’’	*R* ^2^
RD	51,550	0.347	0.994	55,460	0.135	0.965	12,750	0.121	0.984
DCP2	45,830	0.353	0.994	38,780	0.146	0.974	8877	0.125	0.989
DCP6	46,780	0.356	0.994	39,240	0.146	0.975	9570	0.123	0.983
DCP10	41,730	0.401	0.990	34,780	0.175	0.982	9054	0.146	0.979

RD—reference dough; DCP2, DCP6, DCP10—dough with 2, 6 and 10% substitution of starch with cricket powder.

**Table 2:** Results of ^1^H NMR study for dough and bread.

Sample	T_1_ (ms)	T_21_ (ms)	T_22_ (ms)
RD	279.9 ± 3.1 ^A^	5.24 ± 0.88 ^A^	45.46 ± 0.76 ^A^
DCP2	251.9 ± 2.3 ^B^	3.16 ± 0.31 ^B^	43.65 ± 0.56 ^B^
DCP6	246.6 ± 0.9 ^C^	2.25 ± 0.22 ^C^	38.66 ± 0.30 ^C^
DCP10	223.1 ± 0.9 ^D^	2.17 ± 0.35 ^C^	32.10 ± 0.19 ^D^
RB	235.7 ± 1.5 ^a^	1.39 ± 0.22 ^c^	16.07 ± 0.41 ^b^
BCP2	213.4 ± 0.6 ^b^	2.43 ± 0.15 ^a^	16.39 ± 0.31 ^b^
BCP6	198.1 ± 0.8 ^c^	2.52 ± 0.27 ^a^	15.84 ± 0.32 ^b^
BCP10	179.3 ± 0.6 ^d^	2.83 ± 0.25 ^a^	17.05 ± 0.84 ^a^

Mean values denoted by different letters (uppercase for dough, lowercase for bread) differ statistically significantly (*p* < 0.05). NMR—Nuclear Magnetic Resonance; RD—reference dough; DCP2, DCP6, DCP10—dough with 2, 6 and 10% substitution of starch with cricket powder; RB—reference bread; BCP2, BCP6, BCP10—bread with 2, 6 and 10% substitution of starch with cricket powder.

**Table 3:** The results of water activity.

Sample	a_w_ (-)	a_p_ (-)	V_D_ (s^−1^)	V_p_ (s^−1^)
RB	0.925 ± 0.002 ^a^	0.487 ± 0.013 ^a^	0.024 ± 0.002 ^a^	0.0030 ± 0.0001 ^a^
BCP2	0.926 ± 0.003 ^a^	0.503 ± 0.015 ^a^	0.022 ± 0.002 ^ab^	0.0026 ± 0.0001 ^b^
BCP6	0.929 ± 0.006 ^a^	0.641 ± 0.037 ^a^	0.019 ± 0.004 ^b^	0.0025 ± 0.0006 ^b^
BCP10	0.910 ± 0.007 ^a^	0.591 ± 0.016 ^a^	0.019 ± 0.002 ^b^	0.0018 ± 0.0002 ^c^

Mean values denoted by different letters differ statistically significantly (*p* < 0.05). RB—reference bread; BCP2, BCP6, BCP10—bread with 2, 6 and 10% substitution of starch with cricket powder; a_p_—limit water activity (intermediate); a_w_—water activity at equilibrium condition (final); V_D_—transport rate; V_p_—rate of the surface conduction.

**Table 4:** Textural properties of breadcrumbs.

Sample	Hardness (N)	Springiness (%)	Cohesiveness (-)	Chewiness (-)	Resilience (-)
RB	37.21 ± 4.28 ^a^	99.3 ± 1.5 ^a^	0.556 ± 0.022 ^b^	2238 ± 286 ^a^	0.341 ± 0.028 ^b^
BCP2	35.73 ± 1.53 ^a^	99.3 ± 0.5 ^a^	0.612 ± 0.068 ^ab^	2096 ± 277 ^ab^	0.400 ± 0.079 ^a^
BCP6	25.08 ± 2.19 ^b^	99.5 ± 2.2 ^a^	0.645 ± 0.052 ^a^	1726 ± 293 ^b^	0.431 ± 0.035 ^a^
BCP10	24.53 ± 1.79 ^b^	99.9 ± 1.8 ^a^	0.691 ± 0.062 ^a^	1710 ± 77 ^b^	0.443 ± 0.049 ^a^

Mean values denoted by different letters differ statistically significantly (*p* < 0.05). RB—reference bread; BCP2, BCP6, BCP10—bread with 2, 6 and 10% substitution of starch with cricket powder.

## Supplementary Materials

The following are available online at https://www.mdpi.com/2304-8158/8/7/240/s1, Figure S1. Mechanical spectra of gluten-free dough with cricket powder.
