# Supplementary material for: Gluten-Free Bread with Cricket Powder—Mechanical Properties and Molecular Water Dynamics in Dough and Ready Product
Source: Foods. 2019 Jul 3;8(7):240. doi: 10.3390/foods8070240 (PMC6678567; doi:10.3390/foods8070240)
Supplement: Supplementary file 1 [file foods-08-00240-s001.pdf]

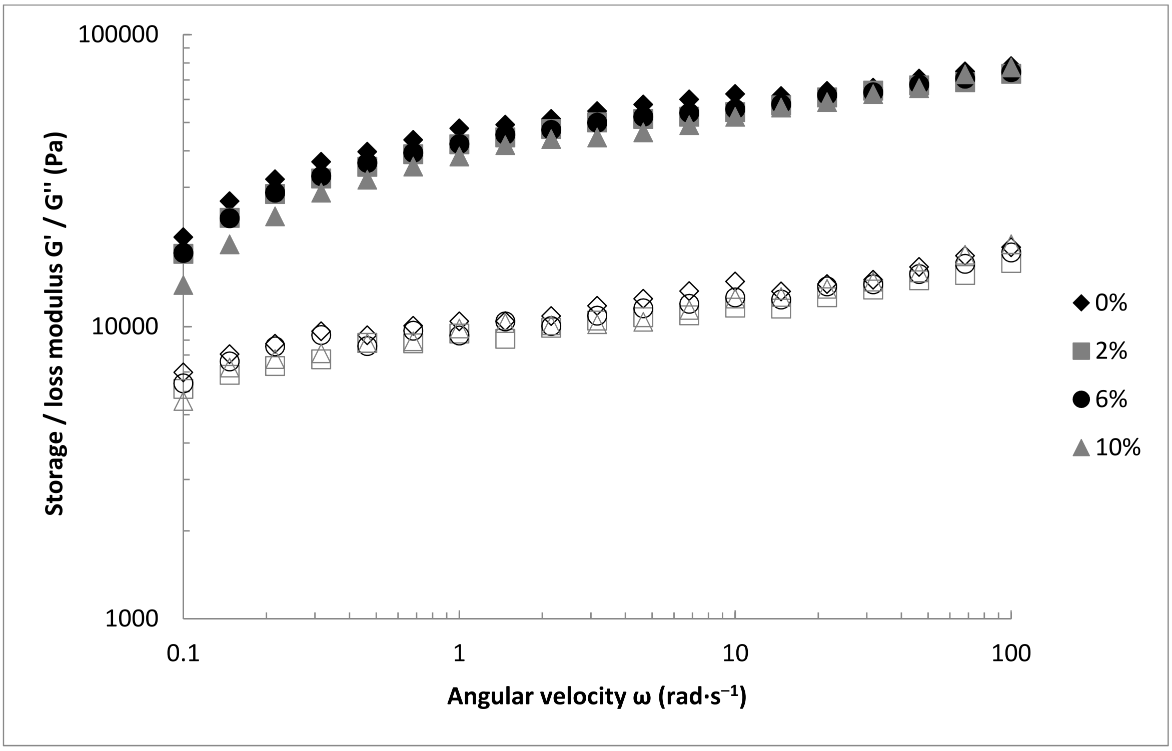

Figure S1. Mechanical spectra of gluten-free dough with cricket powder. Filled markers on the chart present G′ values, while empty markers G′′ values.
